# The antibacterial and antifungal activity of honey and propolis from the Australian stingless bee *Tetragonula carbonaria*

**DOI:** 10.1371/journal.pone.0361610

**Published:** 2026-10-26

**Authors:** Jasmin Li, Rosalyn Gloag, Dee A. Carter, Kenya E. Fernandes

**Affiliations:** 1 School of Life and Environmental Sciences, University of Sydney, Sydney, NSW, Australia; 2 Sydney Infectious Diseases Institute, University of Sydney, Sydney, NSW, Australia; Universiti Teknologi Malaysia - Main Campus Skudai: Universiti Teknologi Malaysia, MALAYSIA

## Abstract

The global rise of antimicrobial-resistant pathogens has increased interest in bee-derived natural products as sources of novel antimicrobials. While most research has focused on European honey bee (*Apis mellifera*) products, the 550 species of stingless bees found globally also produce honey and other hive products, representing a largely unexplored source of novel bioactives. This study investigates the antimicrobial activity of propolis extracts and honey from the Australian stingless bee *Tetragonula carbonaria* against 16 clinically significant bacterial and fungal pathogens, using broth microdilution methodology. *T. carbonaria* propolis and honey exhibited distinct antimicrobial profiles: propolis showed selective activity against a subset of bacteria and certain filamentous fungi, while honey showed broad-spectrum activity against all tested bacteria and selective activity against dermatophyte fungi. The level of H_2_O_2_ production in *T. carbonaria* honey was only moderately correlated with its antimicrobial activity, and the total phenolic content of propolis did not correlate significantly with its antimicrobial activity, suggesting other bee- or plant-derived compounds are important contributors. These findings highlight *T. carbonaria* propolis and honey as promising antimicrobials and potential sources of novel bioactive compounds.

## Introduction

The rapid rise of antimicrobial resistance poses a serious global health threat, with previously treatable infections becoming increasingly difficult to manage. One major challenge in combating antimicrobial resistance is the lengthy and costly process of developing novel antimicrobials. Combined with waning interest from pharmaceutical companies, this has severely limited the pipeline of new treatments [1]. Consequently, there is an urgent need for alternative antimicrobial sources with demonstrated efficacy, and one promising avenue is bee-derived products. Among these are propolis, a mixture of plant resins and beeswax used by bees to build and protect their hives, and honey, a nutrient-rich, sweet substance commonly derived from floral nectar and enzymatically processed by bees. The therapeutic properties of propolis and honey have been recognised since ancient times [2,3], and their use in medical contexts is seeing a modern resurgence due to their broad-spectrum antibacterial and antifungal activity [3,4].

While most modern research has focused on European honey bee products (*Apis mellifera*), there is growing interest in the potential of products from other bee species. Stingless bees (Hymenoptera, Apidae: Tribe Meliponini) are a large, diverse group of social bees, encompassing an estimated 550 species [5]. Honey bees and stingless bees diverged from a common ancestor 70 million years ago, and the two clades of bees exhibit many distinct ecological and behavioural traits [6]. Australia is home to eleven species of stingless bees, including *Tetragonula carbonaria*, a widely managed species that is distributed across tropical, subtropical, and temperate climates around the east coast of Australia. Australian Indigenous communities have long used stingless bee products as a traditional medicine, valuing these both for their therapeutic applications, and for their deep cultural significance, where they play a central role in art, social life, mythology, religion, and spirituality [7,8].

Unlike *A. mellifera* hives, which feature organised wax comb and use propolis primarily to seal gaps and defend against invading insects and pathogens, stingless bee nests consist of small, pot-like propolis structures used to store honey and pollen (Fig 1A) [9]. This distinctive architecture facilitates close contact between honey and the propolis pot walls, and it has been suggested that this may lead to the transfer of bioactive compounds between these materials [10]. The uniqueness of stingless bee products is reflected in their chemical compositions. Propolis from *T. carbonaria* contains a diverse array of plant-derived bioactive compounds including polyphenols, terpenoids, and aromatic acids [11,12]. Similarly, honey from *T. carbonaria* is distinguished from *A. mellifera* honey by a high moisture content (24–28%) and trehalulose as the dominant sugar [13–16].

**Fig 1:**
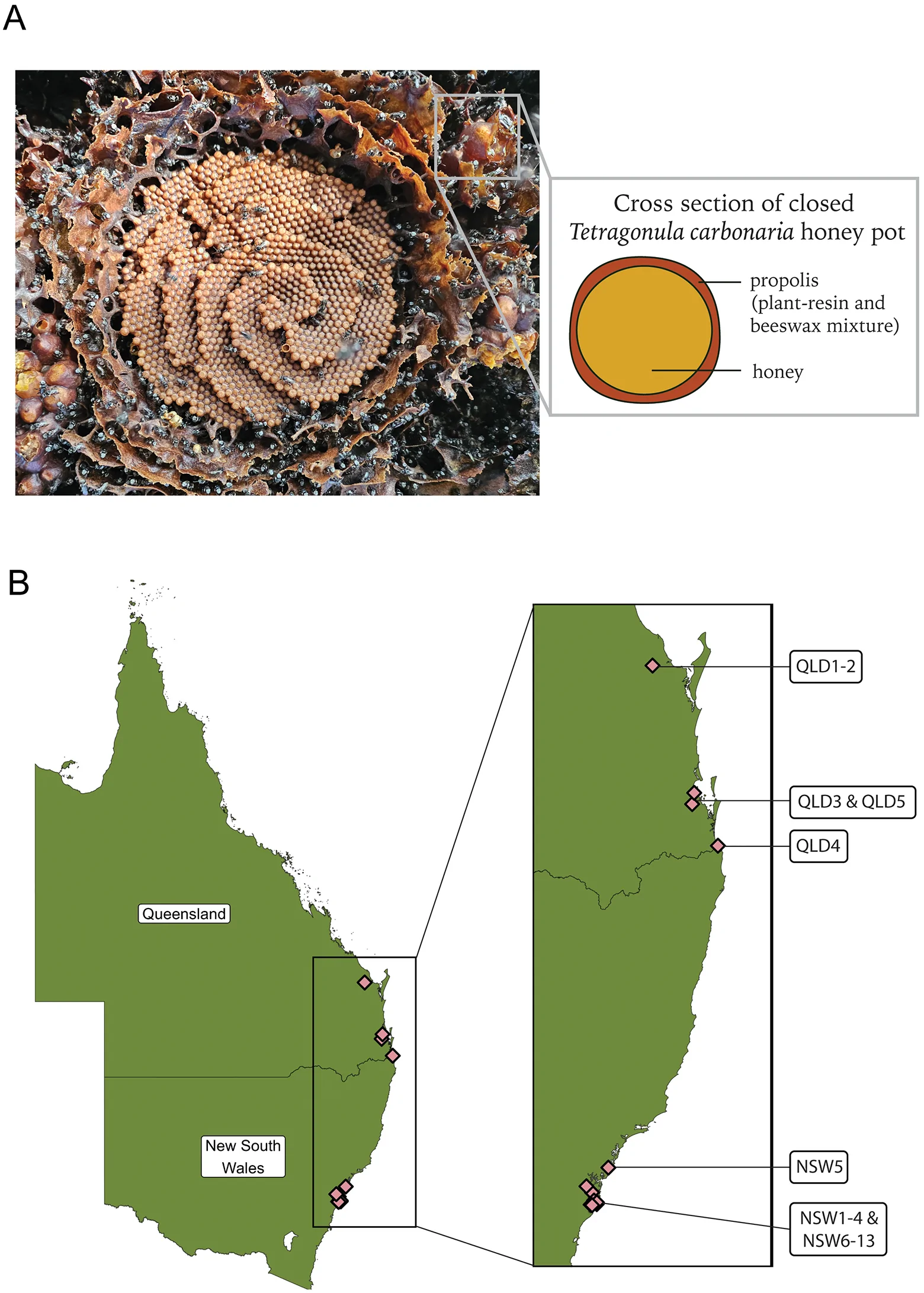
*Tetragonula carbonaria* nests and sampling sites. (A) Internal view of a managed *Tetragonula carbonaria* colony, opened to show the spatial arrangement of nest materials (photograph by Jasmin Li). (B) Geographic location of samples used in this study, created using the *ozmap* package in R v4.5.0 based on Australian Bureau of Statistics data. Markers indicate the area where propolis and/or honey samples were collected across the states of New South Wales (NSW) and Queensland (QLD).

In a previous study, we investigated honey from three different stingless bee species and found that each exhibited distinct antimicrobial mechanisms [17]. Building on this work, the current study aimed to characterise the spectrum of activity of *T. carbonaria* propolis and honey against a diverse set of clinically significant bacterial and fungal pathogens and compare this to simultaneously tested *A. mellifera* products. Our results reveal that *T. carbonaria* propolis and honey exhibit species-specific antimicrobial profiles, with propolis displaying potent but selective antifungal effects, and honey showing broad-spectrum activity against bacteria and certain fungi.

## Materials and methods

### Propolis and honey samples

*Tetragonula carbonaria* propolis and honey samples were sourced from nests across New South Wales (NSW) and Queensland (QLD) between 2021 and 2023, with the assistance of local beekeepers, Dr Alex Austin from the Ku-ring-gai Council Sydney, and members from the Australian Native Bee Association (Fig 1B; S1 Table). All samples came from managed colonies except for two QLD samples (QLD1 and QLD2) which were sampled from wild colonies during their rescue from felled trees. No specific permits were required for this work and sampling was conducted either from privately owned, managed colonies with the owners’ consent or opportunistically during the rescue and relocation of wild colonies from trees that had already been felled for unrelated reasons.

Samples were provided either as intact honey pots (i.e., honey and pot-propolis together) or filtered honey samples (no propolis). The honey and propolis from whole honey pots were separated by splitting open the pots and squeezing the contents into a Falcon tube. The honey was centrifuged at 3,000 x g for 5 min to separate and remove pollen and other debris. After arrival in the lab, all samples were stored in plastic containers at 4 °C in the dark. Comparative reference samples from *Apis mellifera* included one ethanolic propolis extract (AM01) and two commercial antimicrobial honeys – Barnes TA 10+ Jarrah honey (JAR), and Comvita UMF 18+ Manuka honey (MAN).

### Preparation of ethanolic propolis extracts

Ethanolic extracts were prepared from eight propolis samples separated from intact honey pots collected in NSW. Extraction conditions were chosen based on literature reports and pilot experiments to maximise the yield of bioactive compounds [11]. Approximately 1 g of raw propolis per sample was weighed, snap frozen in liquid nitrogen, and crushed to a fine powder using a mortar and pestle. 70% ethanol was added at a ratio of 1:10 (% w/v) propolis to solvent, and tubes were protected from light and set on a shaker at ~50 rpm for 7 days. Tubes were centrifuged to pellet the propolis and the supernatants were transferred into wide mouth samples jars and allowed to evaporate naturally to dryness in a fume hood (approximately 48 hours). The resulting residues were resuspended in DMSO to achieve a final concentration of 100 mg/mL and stored at −30 °C until use.

### Preparation of cultures

The antimicrobial activity of propolis extracts and honey samples was determined against 16 clinically significant microorganisms. These included three Gram-positive bacteria (*Staphylococcus aureus* ATCC29213, *Enterococcus faecalis* ATCC29212, and *Streptococcus pyogenes* MTC03), five Gram-negative bacteria (*Escherichia coli* ATCC25922, *Acinetobacter baumannii* ATCC19606, *Klebsiella pneumoniae* MTC02, *Pseudomonas aeruginosa* ATCC27853, and *Salmonella enterica* MTC01), three yeasts (*Candida glabrata* CBS138, *Candida albicans* SC5314, and *Cryptococcus neoformans* H99), and five moulds (*Aspergillus flavus* ATCC204304, *Aspergillus fumigatus* ATCC204305, *Rhizopus oryzae* ROI01, *Microsporum canis* R236, and *Trichophyton interdigitale* CTI1).

All microorganisms were cultured on agar plates from glycerol stocks except the dermatophytes *M. canis*, and *T. interdigitale,* which were cultured from agar cuts stored in water. Bacterial cultures were incubated on nutrient agar plates at 35 °C for 24 hours. Yeasts and non-dermatophyte moulds were cultured on Potato Dextrose Agar (PDA) and incubated at 30 °C for 24–48 hours or until sporulation. Dermatophyte moulds were cultured on oatmeal agar and incubated at 30 °C for 5 days or until sporulation.

### Antimicrobial susceptibility testing

Minimum inhibitory concentration (MIC) values were determined via broth microdilution assay in 96-well plates following Clinical & Laboratory Standards Institute (CLSI) standard methods for aerobic bacteria, yeasts, and filamentous fungi [18–20].

Inocula were prepared by suspending cells from single colonies on agar plates in 0.85% saline. Bacterial inocula were adjusted to an absorbance of 0.08–0.1 at 625 nm measured using a spectrophotometer. Fungal inocula were counted using a haemocytometer. Inocula were further diluted in the appropriate culture media to obtain final concentrations of 2 x 10^5^–8 x 10^5^ CFU/mL for bacteria, 0.5 x 10^3^–2.5 x 10^3^ CFU/mL for yeasts, 0.4 x 10^4^–1 x 10^4^ CFU/mL for non-dermatophyte moulds, and 1 x 10^3^–3 x 10^3^ CFU/mL for dermatophyte moulds.

Culture media used were Mueller-Hinton Broth (MHB; Oxoid) supplemented with 20 mg Ca^++^ /L and 10 mg Mg^++^ /L for bacteria, Yeast Nitrogen Base (YNB) supplemented with 0.165 M MOPS and 0.5% D-glucose for *Cryptococcus* species, and RPMI-1640 supplemented with 0.165 M MOPS and 2% D-glucose for *Candida* species and moulds.

Honey samples were diluted to 50% (w/v) in Milli-Q water and filter sterilised using 0.22 µm pore filters. Honey samples were then further diluted and assayed at final concentrations of 0.8–25% (w/v). Propolis extracts were diluted in Milli-Q water and initially assayed at final concentrations of 32–1000 µg/mL, followed by additional assays at 1–32 µg/mL against *E. faecalis, S. aureus, E. coli, R. oryzae, T. interdigitale,* and *M. canis*. Sterility controls, growth controls, and drug controls were included for all assays. Tetracycline was used for bacteria and amphotericin B for fungi, at final concentrations of 0.03–64 µg/mL and 0.0005–1 µg/mL, respectively.

Plates were incubated at 35 °C for 18–20 hours (bacteria and *Candida*), 48 hours (*Cryptococcus,* and non-dermatophyte moulds), or 5 days (dermatophyte moulds). For honey, MIC values were determined as the lowest concentration with no visible growth. For propolis extracts, due to the opacity of the samples, 20 µL of 0.15 mg/mL resazurin dye solution was added to each well and colour change from blue to pink was used to determine the presence of viable cells. At least two biological replicates were performed for each experiment until a mode was obtained.

### Measuring H_2_O_2_ concentration in honey samples

Hydrogen peroxide (H_2_O_2_) concentration in honey samples was measured using low-level peroxide test strips (Precision Laboratories Cat. No. IS124-50S), which provide a rapid and simple approach. Previous work with *A. mellifera* honey has shown that measurements at 2 hours using strips correlate well with enzymatic assays measuring peak H_2_O_2_ production, and total H_2_O_2_ production [21]. Samples were initially diluted to 50% (w/v) in deionised water and left to sit for no more than 10 min before further dilution to 25% (w/v) and incubation at room temperature for 30 min. A 30 min incubation was chosen because stingless bee honey has a higher water content than *A. mellifera* honey, which was hypothesised to accelerate peroxide formation, and initial testing showed no significant difference in H_2_O_2_ readings between 30 min and 2 hours. Strips were immersed into the diluted honey solutions and allowed to develop for 10 seconds. The resulting colour change was compared to the colour chart provided by the manufacturer to determine H_2_O_2_ concentration as 0, 3, 10, 30, or 100 parts per million (ppm). A single measurement was taken for each sample.

### Measuring phenolic and antioxidant content in propolis extracts

Total phenolic content was measured using the Folin-Ciocalteu (FC) assay, and antioxidant capacity was measured using the ferric-reducing antioxidant power (FRAP) assay, following previously published methods [21] modified for propolis. Briefly, ethanolic propolis extracts were prepared at 1 mg/mL and analysed in triplicate using the FC and FRAP assays, with gallic acid and FeSO_4_ used as standards, respectively. Colour controls were included to account for interference from the inherent colour of the propolis extracts. Absorbance was measured at 760 nm for the FC assay and 594 nm for the FRAP assay, and results were calculated from the respective standard curves and expressed as mg gallic acid equivalents per g propolis (mg GAE/g) and μmol Fe²⁺ per g propolis (μmol Fe² ⁺ /g), respectively.

### Statistical analysis

All statistical analyses were performed using GraphPad Prism v10.0.2 (GraphPad Inc.). The normality and lognormality of the data distributions were first assessed using the d’Agostino & Pearson test. MIC values were log-transformed prior to further analysis unless stated otherwise. For propolis extract data, repeated measures ANOVA followed by Tukey’s multiple comparisons test was used to compare the MIC values across all microbial species. Pearson correlation was used to assess the consistency of microbial susceptibility across different propolis extracts.

For correlations between H_2_O_2_ and antimicrobial activity, MIC values were subtracted from 25 so that variables related to increased antimicrobial activity would appear positively, rather than negatively, correlated. Spearman’s rank correlation was then used to assess the relationship between the reversed MIC values and H_2_O_2_ concentration. For correlations between total phenolic content, antioxidant reducing capacity, and antimicrobial activity, MIC values were similarly subtracted from 1000 to reverse the direction of the scale. Phenolic and antioxidant measurements were log-transformed, and Pearson correlation was used to assess their relationships with the reversed MIC values.

## Results

### *T. carbonaria* propolis exhibits highly selective antimicrobial activity across samples

The antimicrobial activity of eight *T. carbonaria* ethanolic propolis extracts was tested against a range of clinically relevant bacteria and fungi. MIC distributions are shown in Fig 2 with individual MIC values summarised in Table 1. There was a high level of consistency among the extracts, showing uniformly inactive (MIC > 1000 µg/mL) or uniformly active profiles against each microbial species. Where activity was observed, extracts showed greater variability in MIC values against susceptible bacteria than against susceptible fungi.

**Table 1:** Antimicrobial activity of *Tetragonula carbonaria* propolis extracts.

Species Tested	Minimum Inhibitory Concentration (µg/mL)^1^								
	NSW1	NSW2	NSW3	NSW4	NSW5	NSW6	NSW7	NSW8	AM01^2^
**Gram-positive bacteria**									
*Staphylococcus aureus*	32	8	32	8	32	8	4	8	8
*Enterococcus faecalis*	128	32	128	32	128	16	8	16	8
*Streptococcus pyogenes*	>1000	>1000	>1000	>1000	>1000	>1000	>1000	>1000	>1000
**Gram-negative bacteria**									
*Escherichia coli*	128	8	32	8	32	32	16	32	8
*Acinetobacter baumannii*	>1000	>1000	>1000	>1000	>1000	>1000	>1000	>1000	>1000
*Klebsiella pneumoniae*	>1000	>1000	>1000	>1000	>1000	>1000	>1000	>1000	>1000
*Pseudomonas aeruginosa*	>1000	>1000	>1000	>1000	>1000	>1000	>1000	>1000	>1000
*Salmonella enterica*	>1000	>1000	>1000	>1000	>1000	>1000	>1000	>1000	>1000
**Yeasts**									
*Candida glabrata*	>1000	>1000	>1000	>1000	>1000	>1000	>1000	>1000	<32
*Candida albicans*	>1000	>1000	>1000	>1000	>1000	>1000	>1000	>1000	<32
*Cryptococcus neoformans*	>1000	>1000	>1000	>1000	>1000	>1000	>1000	>1000	<32
**Moulds**									
*Aspergillus flavus*	>1000	>1000	>1000	>1000	>1000	>1000	>1000	>1000	<32
*Aspergillus fumigatus*	>1000	>1000	>1000	>1000	>1000	>1000	>1000	>1000	<32
*Rhizopus oryzae*	16	32	32	64	64	32	32	32	16
*Microsporum canis*	16	16	16	32	32	32	16	16	2
*Trichophyton interdigitale*	16	16	32	32	32	32	32	32	4

1 < 32 and >1000 indicates the value of the MIC fell outside of the range of testing.

^2^*Apis mellifera* propolis included for comparison.

**Fig 2:**
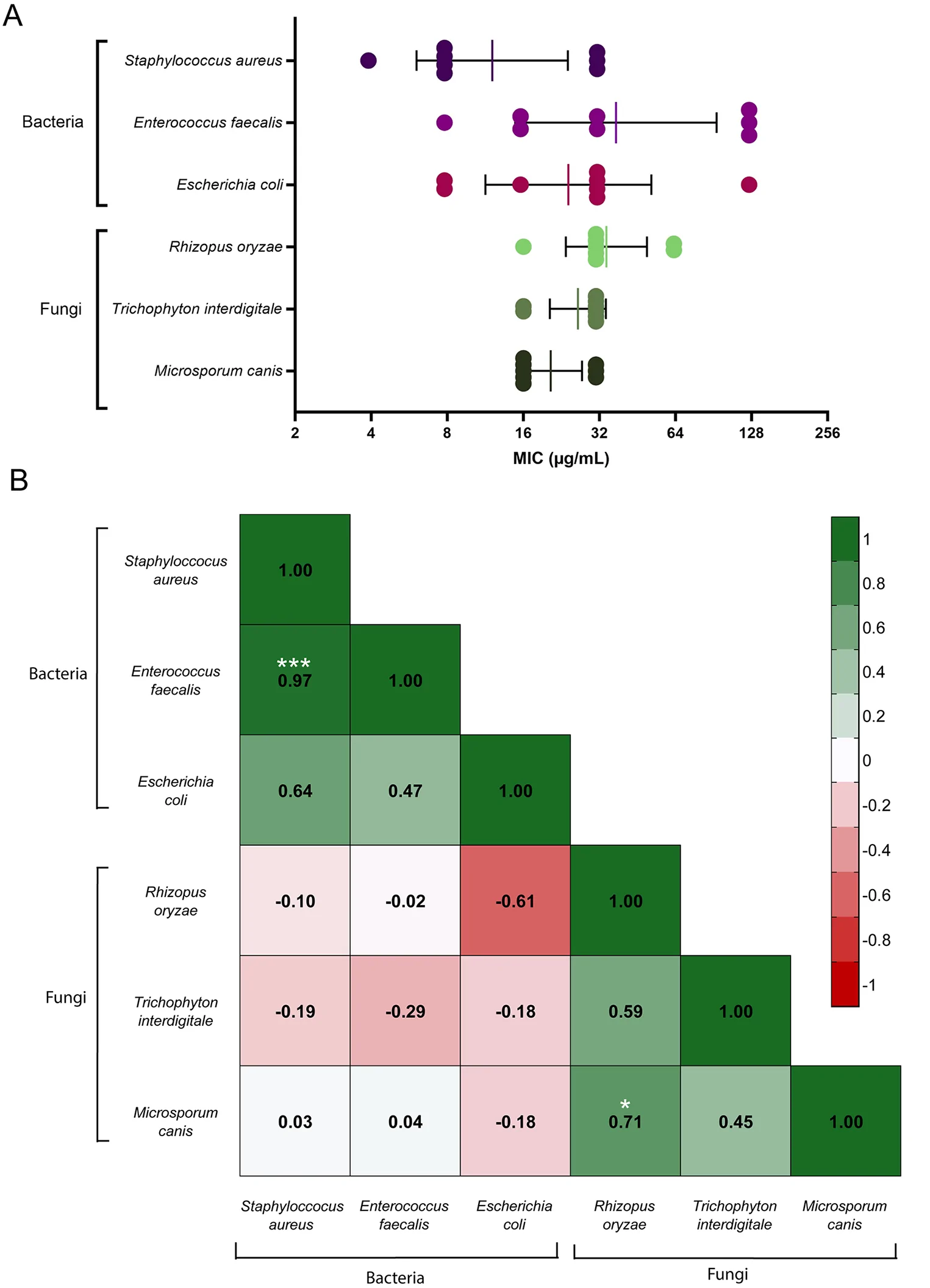
Antimicrobial activity of *T. carbonaria* propolis extracts against susceptible bacteria and fungi. (A) The distribution of MIC values for propolis extracts (n = 8) against susceptible bacterial and fungal species. Dots represent the MICs of individual samples and coloured vertical lines show the geometric mean with error bars showing the 95% CI. (B) Heatmap showing Pearson correlation coefficients between MIC values for propolis extracts across susceptible bacterial and fungal species. Correlation coefficients are displayed within each cell, with significance levels indicated above. *** p < 0.001; * p < 0.05.

Propolis extracts were active against the Gram-positive bacteria *S. aureus* and *E. faecalis* and the Gram-negative bacterium *E. coli,* but had no detectable activity against the Gram-positive bacterium *S. pyogenes* and the Gram-negative bacteria *A. baumannii, K. pneumoniae, P. aeruginosa,* and *S. enterica* (MICs > 1000 µg/mL). The *A. mellifera* propolis extract (AM01), included here for comparison, had an identical antibacterial spectrum. Antibacterial MIC values for *T. carbonaria* propolis extracts ranged from 4–128 µg/mL, with *S. aureus* being most susceptible (geomean = 12 µg/mL), followed by *E. coli* (geomean = 24 µg/mL), and *E. faecalis* (geomean = 37 µg/mL) (Fig 2A).

Considering antifungal activity, propolis extracts were active against the moulds *R. oryzae, M. canis,* and *T. interdigitale,* but had no detectable activity against the yeasts *C. glabrata, C. albicans* and *C. neoformans,* or against the moulds *A. flavus and A. fumigatus* (MICs > 1000 µg/mL) (Table 1). The *A. mellifera* propolis extract AM01 had a notably different antifungal activity profile compared to the *T. carbonaria* propolis extracts, being active against all tested yeast and mould species. Antifungal MIC values for *T. carbonaria* propolis extracts ranged from 16–64 µg/mL with *M. canis* being most susceptible (geomean = 20 µg/mL), followed by *T. interdigitale* (geomean = 26 µg/mL), and *R. oryzae* (geomean = 34 µg/mL) (Fig 2A).

Overall, there was no significant difference in average susceptibility levels between bacterial and fungal species (only including MICs < 1000 µg/mL; *p* > 0.05), but bacterial species had significantly more variable MIC values (*p* < 0.001). Comparing the susceptibility profile of each microbe to individual propolis extracts, activity between different bacterial species was generally positively correlated, reaching significance for *S. aureus* and *E. faecalis* (*r* = 0.97; *p* < 0.001). Activity between different fungal species was also generally positively correlated, reaching significance for *R. oryzae* and *M. canis* (*r* = 0.71; *p* = 0.05) (Fig 2B). Activity between fungal and bacterial species was generally negatively correlated, though not significantly for any species pair, suggesting the pool of compounds underlying antifungal activity may be distinct from those driving antibacterial activity.

To investigate potential compounds and drivers behind the antimicrobial activity of propolis extracts, we examined their total phenolic content and antioxidant reducing capacity. Total phenolic content ranged from 30.0–61.1 mg GAE/mg, and antioxidant reducing capacity from 241.1–771.1 µmol Fe^2+^/g (Fig 3). The two parameters were strongly correlated (r = 0.85; p < 0.001), indicating that extracts with higher phenolic content also tended to have greater antioxidant reducing capacity. However, neither parameter was significantly correlated with MIC values for susceptible bacteria or fungi (p > 0.05). Thus, variation in total phenolic content and antioxidant reducing capacity did not explain variation in antimicrobial activity among the propolis extracts.

**Fig 3:**
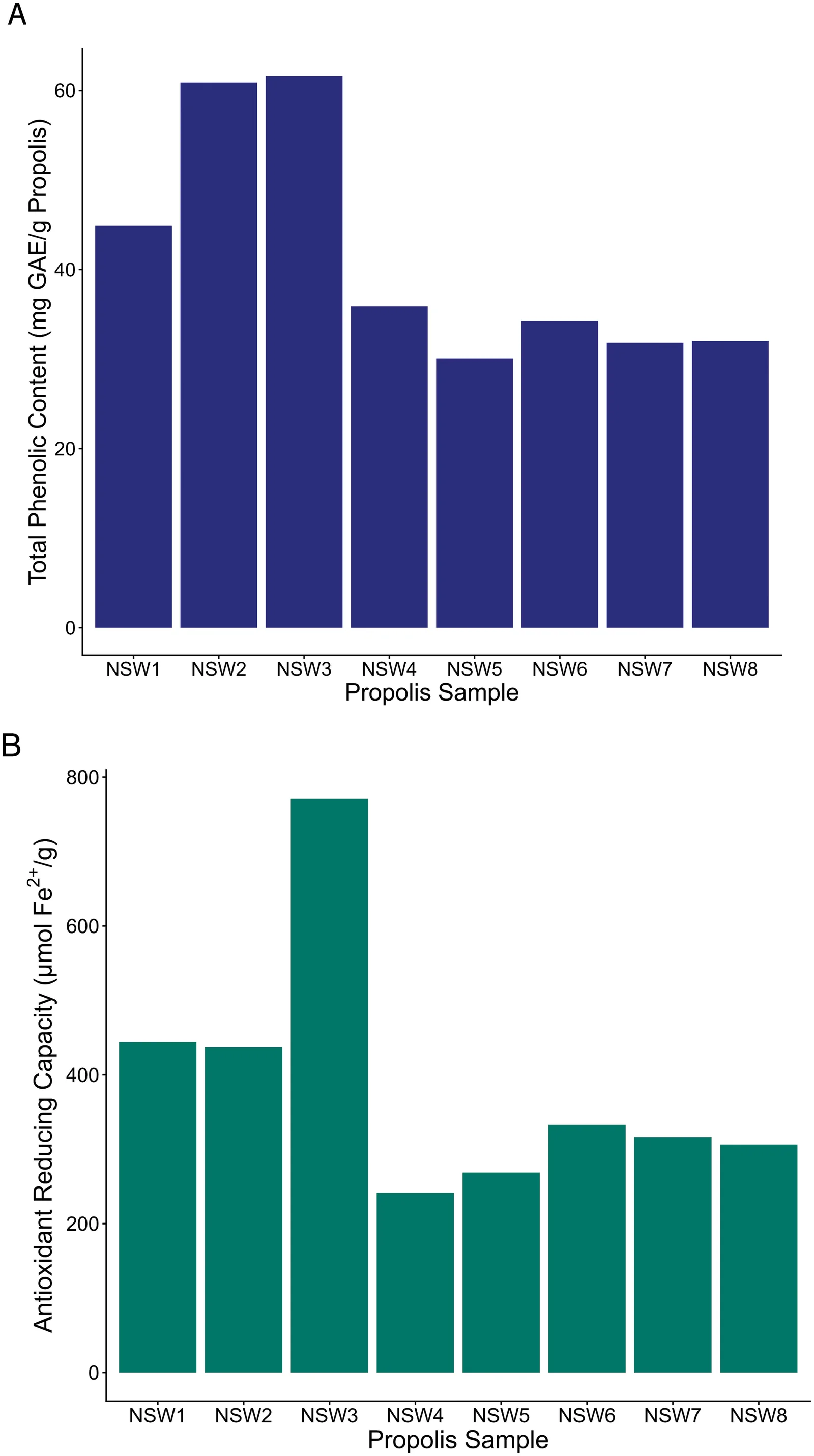
Total phenolic content and antioxidant reducing capacity of *T. carbonaria* propolis extracts. Bar charts displaying (A) total phenolic content measured by the Folin-Ciocalteu (FC) assay using gallic acid as the standard, and (B) antioxidant reducing capacity measured by the ferric-reducing antioxidant power (FRAP) assay using FeSO_4_ as the standard.

### *Tetragonula carbonaria* honey exhibits broad-spectrum antibacterial but selective antifungal activity

Seven *T. carbonaria* honey samples were initially screened against four representative bacteria and fungi of clinical concern: *S. aureus, E. coli, C. glabrata* and *A. flavus.* MIC distributions are shown in Fig 4, with individual MIC values summarised in Table 2. All of the honey samples were active against the bacteria *S. aureus* and *E. coli* but had no detectable activity against the fungi *C. glabrata* and *A. flavus* (MICs > 25% w/v). There was no significant difference in susceptibility levels between *S. aureus* (geomean = 5.1% (w/v)) and *E. coli* (geomean = 4.2% (w/v)).

**Table 2:** Antimicrobial activity of *Tetragonula carbonaria* honeys.

Species Tested	Minimum Inhibitory Concentration (%(w/v))^1,2^								
	NSW2	NSW3	NSW4	NSW5	NSW6	NSW7	NSW8	MAN^3^	JAR^3^
**Gram-positive bacteria**									
*Staphylococcus aureus*	6	6	6	6	3	6	3	6	6
*Enterococcus faecalis*	–	–	–	25	13	13	–	13	25
*Streptococcus pyogenes*	–	–	–	13	6	6	–	6	6
**Gram-negative bacteria**									
*Escherichia coli*	3	6	6	6	3	3	3	6	3
*Acinetobacter baumannii*	–	–	–	13	6	6	–	6	6
*Klebsiella pneumoniae*	–	–	–	25	13	13	–	13	13
*Pseudomonas aeruginosa*	–	–	–	25	6	6	–	25	13
*Salmonella enterica*	–	–	–	25	13	13	–	6	13
**Yeasts**									
*Candida glabrata*	>25	>25	>25	>25	>25	>25	>25	>25	>25
*Candida albicans*	–	–	–	>25	>25	>25	–	>25	>25
*Cryptococcus neoformans*	–	–	–	>25	25	25	–	>25	13
**Moulds**									
*Aspergillus flavus*	>25	>25	>25	>25	>25	>25	>25	>25	>25
*Aspergillus fumigatus*	–	–	–	>25	>25	>25	–	25	25
*Rhizopus oryzae*	–	–	–	>25	25	>25	–	>25	25
*Microsporum canis*	–	–	–	6	2	3	–	2	<1
*Trichophyton interdigitale*	–	–	–	25	3	3	–	3	<1

^1^Dashes (-) indicate that the honey sample was not tested against that organism due to insufficient sample volume.

^2^< 1 and >25 indicates the value of the MIC fell outside of the range of testing.

^3^Active *Apis mellifera* honeys included for comparison. MAN = Comvita UMF 18+ Manuka honey. JAR = Barnes TA 10+ Jarrah honey.

**Fig 4:**
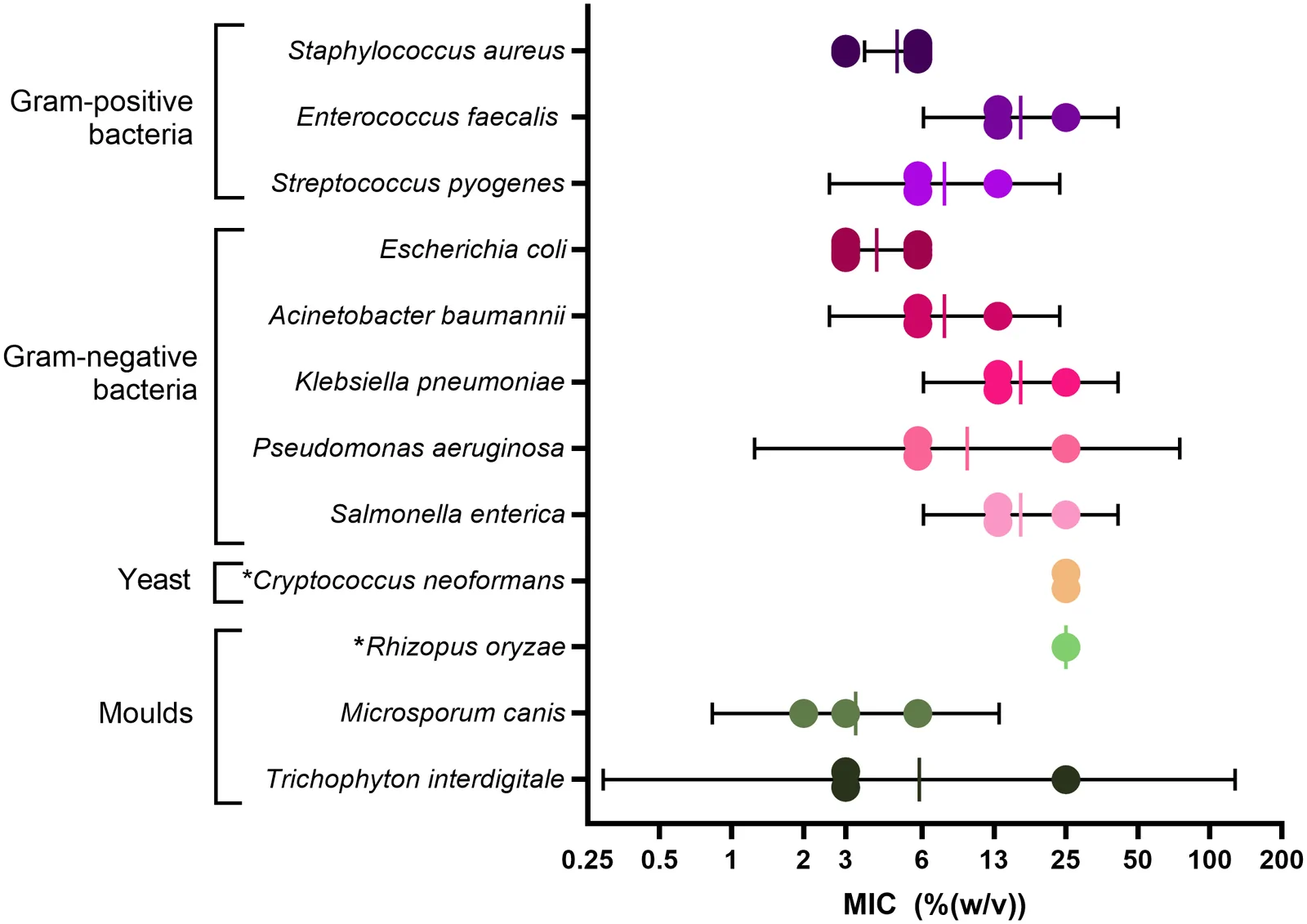
Antimicrobial activity of *T. carbonaria* honey against susceptible bacteria and fungi. The distribution of honey samples from NSW with achievable MIC values within the tested range of 0.8-25% (w/v). Dots represent the mode MIC value for each sample and coloured vertical lines show the geometric mean with error bars showing the 95% CI. An asterisk (*) indicates the exclusion of samples that had MIC values >25% (w/v).

Due to limited sample volume, a subset of three honey samples (NSW5, NSW6 and NSW7) were selected for further antimicrobial testing against a wider panel of clinically significant human pathogens (Fig 4; Table 2). These honey samples were active against all tested bacteria: the Gram-positive bacteria *S. aureus, E. faecalis* and *S. pyogenes*, and the Gram-negative bacteria *E. coli, A. baumannii, K. pneumoniae, P. aeruginosa* and *S. enterica* (Fig 4). The antifungal profiles of the honey samples were more mixed. All were active against the dermatophytes *M. canis* and *T. interdigitale*, some were active against the yeast *C. neoformans* and the mould *R. oryzae*, and all were inactive against the yeasts *C. glabrata* and *C. albicans*, and the moulds *A. flavus* and *A. fumigatus.*

To explore potential drivers of antimicrobial activity, we examined H_2_O_2_ production, a key contributor to honey antimicrobial activity (Fig 5). Most samples had a reading of 30 ppm H_2_O_2_, although a few were lower (3–10 ppm). A moderately significant correlation was found between H_2_O_2_ concentration and antimicrobial activity against *S. aureus* (*r* = 0.58; *p* = 0.01), but not *C. neoformans* (*p* > 0.05) suggesting that while H_2_O_2_ contributes to activity, additional bee- or plant-derived compounds are likely to play significant roles in the observed antimicrobial potency.

**Fig 5:**
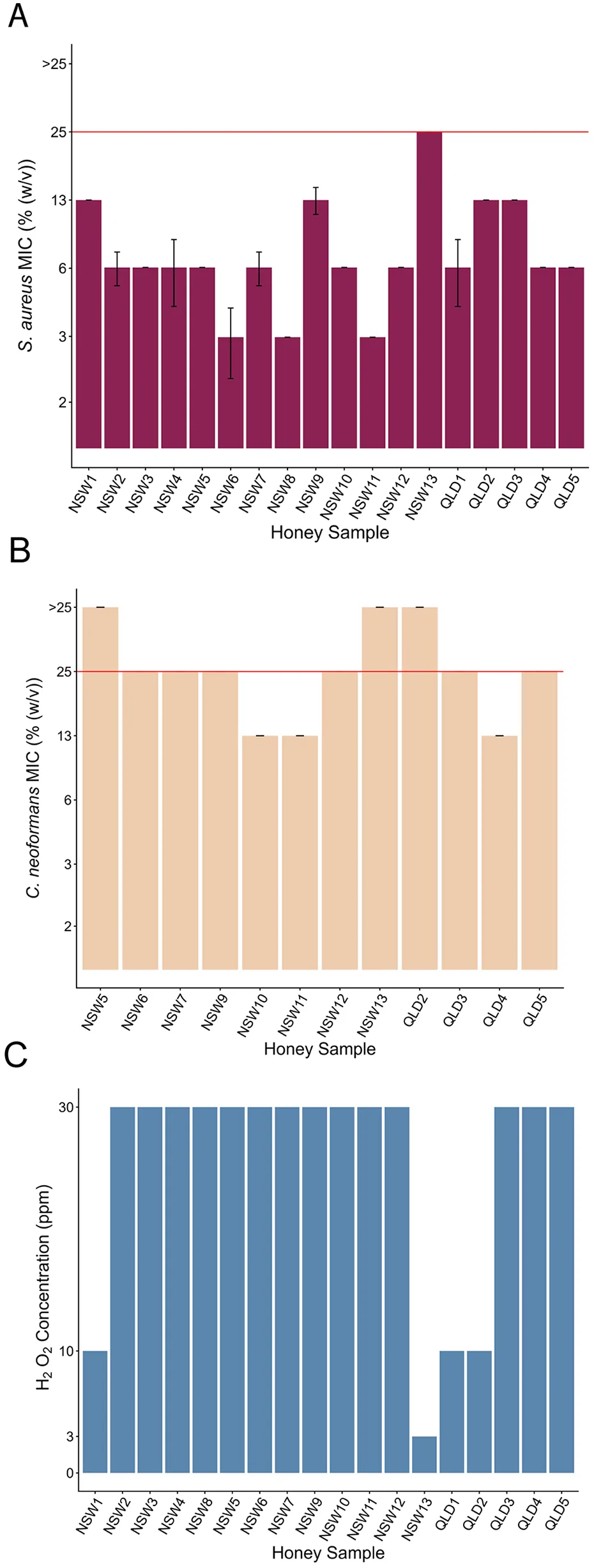
The antimicrobial profiles and hydrogen peroxide concentrations of *T. carbonaria* honey samples. The antimicrobial activity of honey samples against (A) *S. aureus* and (B) *C. neoformans*. Columns indicate the mode with error bars showing SEM. Solid red lines indicate the highest concentration of honey tested, with samples below having detectable antimicrobial activity. (C) H_2_O_2_ concentration assessed using test strips in honey samples after dilution to 25% (w/v) and incubation for 30 minutes.

## Discussion

Previous studies have identified *T. carbonaria* honey and propolis as promising therapeutic candidates due to their strong antimicrobial activity [10,13,17]. However, their effectiveness against a broader spectrum of clinically significant pathogens, particularly fungi, has yet to be determined. In this study, we tested *T. carbonaria* propolis extracts and honey against 16 medically important bacterial and fungal pathogens. We found that *T. carbonaria* propolis and honey exhibited distinct antimicrobial profiles that were different to the corresponding products from *A. mellifera.* In particular, *T. carbonaria* propolis showed a narrow but potent antibacterial and antifungal spectrum, with no activity against yeasts and selective inhibition of filamentous fungi, while *T. carbonaria* honey showed broad antibacterial activity and selective antifungal activity, with some samples demonstrating activity against yeasts and filamentous fungi.

### The antimicrobial profiles of *Tetragonula carbonaria* propolis and honey are specific and distinct from each other

The ethanolic extracts of *T. carbonaria* propolis inhibited a similar spectrum of bacteria to *A. mellifera* propolis. Both propolis types were active against *S. aureus, E. faecalis,* and *E. coli* and were inactive (MIC > 1000 µg/mL) against *S. pyogenes, A. baumannii, K. pneumoniae, P. aeruginosa,* and *S. enterica*. Previous studies using methanolic extraction methodology found *T. carbonaria* propolis from eastern Australia had similar MICs against *S. aureus* to those reported here [10], but *A. mellifera* propolis had much higher MICs (400–2000 µg/mL) [10,22]. This aligns with a global analysis of 324 *A. mellifera* ethanolic propolis extracts, which reported average MICs of 475 µg/mL against *S. aureus,* 544 µg/mL against *Enterococcus sp.,* and 784 µg/mL against *E. coli* [23]. In contrast, the ethanolic *A. mellifera* propolis extract tested in this study (AM01), which was a representative *A. mellifera* propolis sourced from the NSW area, had an MIC of 8 μg/mL for all three bacterial species, similar to the most active *T. carbonaria* extracts. These substantial differences may reflect variations in extraction methodology (e.g., crushing method, solvent exposure time, evaporation process), regional flora, or other environmental factors. To determine whether *T. carbonaria* consistently produces potent antimicrobial propolis, further testing against the same panel of pathogens is required with a broader set of *A. mellifera* samples collected from the same region and processed under identical conditions. The above global analysis additionally reported that *A. mellifera* ethanolic propolis extracts had quantified average MICs exceeding 1000 µg/mL against *A. baumannii, K. pneumoniae*, *P. aeruginosa*, and *Salmonella sp.* However, while activity can be seen at these very high concentrations, they are unlikely to be relevant either ecologically or therapeutically [23].

The antifungal activity of *T. carbonaria* propolis was highly selective, being very active against *M. canis* and *T. interdigitale* and having limited activity against *R. oryzae* and no activity against *A. flavus, A. fumigatus* and all tested yeast species (*C. glabrata, C. albicans, C. neoformans*)*.* In contrast, *Apis mellifera* propolis extracts, including the one tested here, are generally active against a broader range of fungi [11,24,25]. This species-specific but potent antifungal activity of *T. carbonaria* propolis is unusual even among stingless bees, as propolis produced by other stingless bee species has shown activity against *C. albicans* (range of MICs = 256–512 µg/mL) [26]. These findings suggest that *T. carbonaria* propolis possesses a more targeted antifungal profile compared to propolis produced by *A. mellifera* and by other stingless bee species. This selective potency may reflect differences in the types of bioactive plant resins collected by *T. carbonaria*, or in the microbial communities present in their nesting environments [27]. While the dermatophytes tested are primarily human-associated, the compounds responsible have likely evolved to deter environmental filamentous fungi, with activity against human pathogens being incidental. Overall, *T. carbonaria* propolis represents a promising source of antifungal compounds with specificity for certain moulds.

*Tetragonula carbonaria* honey demonstrated a broad antimicrobial profile, with MICs comparable to those of medicinal-grade Manuka and Jarrah honey. Like *A. mellifera* honey, it had stronger activity against bacteria and dermatophytes than against yeasts and *Aspergillus* spp [21,28]. However, unlike *A. mellifera* honey, which is known to exhibit substantial variation in antimicrobial potency between samples, the activity of *T. carbonaria* honey appears to be more consistent across samples [13,17]. Recent findings in other insect-derived honeys, such as the highly species-specific antimicrobial activity of Australian native honeypot ant honey [29], highlight the evolutionary diversity of antimicrobial strategies in eusocial insects. The broad-spectrum activity observed in *T. carbonaria* honey adds to this growing body of evidence, reinforcing native species as promising sources of novel antimicrobials. In the case of whole *T. carbonaria* honey, it may have potential for therapeutic use against bacteria and dermatophytes, while *T. carbonaria* propolis could be a source of antimicrobial leads rather than a broad-spectrum agent.

### Potential sources of antimicrobial activity in *T. carbonaria* propolis and honey

We examined the role of H_2_O_2_, a well-established antimicrobial mechanism in *A. mellifera* honeys, and found that it was present in *T. carbonaria* honey but was only moderately correlated with antibacterial activity, suggesting that other components are at play. In propolis, we found that total phenolic content and antioxidant reducing capacity were strongly correlated with each other, but neither was associated with antimicrobial activity. While overall phenolic content and antioxidant reducing capacity do not explain variation in antimicrobial activity, specific phenolic compounds with antimicrobial mechanisms unrelated to antioxidant activity, or synergistic interactions between compounds, may still contribute.

The similar antimicrobial potency across *T. carbonaria* propolis and honey samples suggests that the key bioactive components likely stem from stable sources, such as bee-derived peroxide production, bee-derived secretions, or selective plant foraging. Massaro and colleagues (2014) identified flavanones originating from the fruit resin of *Corymbia torelliana* as key antibacterial constituents in Queensland propolis produced from *T. carbonaria* [10]. However, *C. torelliana* is native to only a narrow region of northeastern Queensland and has a patchy introduced range in southeastern Queensland and northern New South Wales [30]. This limited range suggests that *C. torelliana*-derived flavanones may contribute to propolis activity only in some regions, and that other plant sources may be responsible for the comparable antimicrobial potency observed in propolis collected elsewhere.

Our previous work using High-Performance Liquid Chromatography – Mass Spectrometry (HPLC-MS/MS) found distinct protein profiles in *T. carbonaria* honey compared to *A. mellifera* honey [17], raising the possibility that in addition to phytochemicals, bee-derived antimicrobial peptides could play a role in honey activity. This is supported by reports of antimicrobial cuticular secretions in *T. carbonaria* [31], which may contribute to the chemical defence of hive products.

### The antifungal activities of propolis and honey may shape the fungal communities in *T. carbonaria* hives

The narrow and potent antifungal activity of *T. carbonaria* propolis and honey raises intriguing questions about the ecological role of these compounds and their evolutionary origin, particularly in shaping the fungal community within the hive. *Tetragonula carbonaria* behaviour and hive structure differ remarkably from *A. mellifera,* and their hive products likely serve distinct ecological functions. Unlike *A. mellifera, T. carbonaria* hives are built predominantly from propolis rather than beeswax, and the ratio of beeswax to plant resin varies across different nest structures [7]. The entrance, for example, contains a higher proportion of plant resin, creating a rigid barrier that deters pests and limits pathogen establishment [7]. In contrast, the food pots contain more beeswax, making them malleable enough for shaping and for easy access to stored honey. However, this reduced concentration of plant-derived phenolics may come at the cost of increased susceptibility to fungal proliferation, which is exacerbated by the relatively high water content of *T. carbonaria* honey and the relatively narrow antifungal spectrum of *T. carbonaria* propolis and honey. Given growing evidence that stingless bees depend on specific yeast associates and yeast metabolites for development [32,33], this selectivity may reflect evolutionary pressures to inhibit harmful filamentous fungi in their food stores while permitting beneficial taxa, a hypothesis that warrants further investigation.

## Limitations & conclusions

While our findings demonstrate that *T. carbonaria* honey and propolis possess attractive antimicrobial properties for further therapeutic development, some limitations must be acknowledged. Our sample set, although geographically distributed, was relatively small and centred primarily around Sydney in New South Wales (Fig 1B). *T. carbonaria* ranges widely along the east coast of Australia, so there may be unobserved regional differences in chemistry due to floral availability or climatic conditions. A closer examination of the floral sources for each sample may reveal finer-scale variation and the compounds associated with its antimicrobial activity.

Despite these limitations, our findings show that both *T. carbonaria* propolis and honey are promising sources of antimicrobial agents, with distinct mechanisms of action compared to propolis and honey produced by *A. mellifera,* and a consistent level of potency. While direct therapeutic use of the honey may be constrained by limited production volume (estimated at around 1 kg of honey per hive annually [34]), these materials nonetheless represent valuable leads for the identification of novel bioactive compounds. Collectively, our findings support increased investigation of native bee species as underexplored sources of antimicrobial agents relevant to combating problematic human pathogens.

## Supporting information

S1 Table Sample collection location and dates. (DOCX)
