# Supplementary material for: The antibacterial and antifungal activity of honey and propolis from the Australian stingless bee Tetragonula carbonaria
Source: PLoS One. 2026 Oct 26;21(10):e0361610. doi: 10.1371/journal.pone.0361610 (PMC13704853; doi:10.1371/journal.pone.0361610)
Supplement: Supplementary file 1 [file pone.0361610.s001.docx]

| **Sample ID** | **Location** | **Date of Collection** |
| --- | --- | --- |
| NSW1 | Ku-ring-gai, Sydney | October, 2022 |
| NSW2 | Ku-ring-gai, Sydney | October, 2022 |
| NSW3 | Ku-ring-gai, Sydney | October, 2022 |
| NSW4 | Ku-ring-gai, Sydney | October, 2022 |
| NSW5 | Central coast | December, 2022 |
| NSW6 | University of Sydney, Sydney | February, 2023 |
| NSW7 | University of Sydney, Sydney | February, 2023 |
| NSW8 | University of Sydney, Sydney | February, 2023 |
| NSW9 | Bexley, Sydney | October, 2021 |
| NSW10 | Maroubra, Sydney | October, 2021 |
| NSW11 | Randwick, Sydney | October, 2021 |
| NSW12 | Brighton-Le-Sands, Sydney | October, 2021 |
| NSW13 | Galston, Sydney | 2023 |
| QLD1 | Childers | 2023 |
| QLD2 | Childers | 2023 |
| QLD3 | Brisbane | 2023 |
| QLD4 | Tugun, Gold Coast | 2022 |
| QLD5 | Brighton, Brisbane | 2022 |

**Supplementary Table S1: Sample collection location and dates**
