# Supplementary material for: The antibacterial and antifungal activity of honey and propolis from the Australian stingless bee Tetragonula carbonaria
Source: PLoS One. 2026 Oct 26;21(10):e0361610. doi: 10.1371/journal.pone.0361610 (PMC13704853; doi:10.1371/journal.pone.0361610)
Supplement: Supplementary file 2 [file pone.0361610.s003.docx]

We thank the reviewers for their time and constructive feedback which we have used to improve our manuscript. We address each comment below.

**Reviewer #1:**

The present manuscript is dedicated to the antibacterial and antifungal potential of honey and propolis from the Australian stingless bee Tetragonula carbonaria. The results are interesting and important. However, there is a major concern: no chemical characterization of the studied bee products has been performed, only H2O2 levels of honey were determined. The Authors should add chemical profiling of the studied products, to make their interesting results much more valuable and scientifically sound.

We thank the reviewer for raising this important point, and we agree that chemical characterisation adds valuable context to the interpretation of our antimicrobial findings. We would like to clarify that the present manuscript builds directly on our previous work (<https://doi.org/10.1128/aem.02523-24>), in which we conducted detailed physicochemical characterisation of honey from *T. carbonaria* and two other Australian stingless bee species. As this characterisation has already been published by our group and is referenced in the manuscript, we did not consider it necessary to repeat it here (mentioned L70-71).

For propolis, in response to the reviewer's comment we have added chemical profiling by measuring total phenolic content (Folin-Ciocalteu assay) and antioxidant reducing capacity (FRAP assay) and tested these against the observed antimicrobial activity. These additions are reflected in the Abstract (L20-21), Methods (L165-174, L186-190), Results (L242-254) and Discussion (L387-392). We have additionally added a new figure (Figure 3) to present the phenolic content and antioxidant capacity data.

**Reviewer #2:**

Dear authors, please address the following concerns:

1. L93: “Ethanolic extracts were prepared from eight propolis samples from NSW”. Was this referring to intact honey pots sample or filtered honey samples?

Thank you for flagging this ambiguity. The propolis used for ethanolic extraction was separated from intact honey pot samples. We have updated the Methods to state this explicitly (L98-99).

2. How many replicated were prepared for H2O2 concentration test?

A single measurement was performed for the H_2_O_2_ test strip measurements for each honey sample. We have updated the Methods to clarify this (L163-164).

3. L151: H2O2 ; state in full name for first introduction of the acronym.

We have corrected this and now spell out “hydrogen peroxide (H_2_O_2_)” at first mention (L152).

4. L159: symbols missing

We appreciate the reviewer flagging this, but we are unable to identify which specific symbols were missing at this line. We have carefully re-checked the manuscript for formatting and symbol errors in general.

5. What is the SD for results displayed in Table 2?

For honey samples with a discrete (i.e. measurable) MIC value in Table 2, the standard error of the mean is shown in Figure 2. Unfortunately, we are not able to calculate a standard devision for samples where the MIC exceeded the highest tested concentration (>25 % w/v), as no numerical endpoint was reached.

6. Figure 1 (B): It is advisable to change into a proper map.

Figure 1B is a geographically accurate map, generated in R using the ozmap package and Australian Bureau of Statistics data. We have updated the figure caption (L65-66) to make the source and method of the map’s construction clearer.

7. Look into typos and grammatical errors meticulously.

We thank the reviewer for this note and have carefully re-proofread the entire manuscript, correcting small typos and grammatical errors.

**Journal Requirements:**

1. Please ensure that your manuscript meets PLOS ONE's style requirements, including those for file naming.

The manuscript conforms to the PLOS ONE formatting and style requirements.

2. In your Methods section, please provide additional information regarding the permits you obtained for the work. Please ensure you have included the full name of the authority that approved the field site access and, if no permits were required, a brief statement explaining why.

Additional information has been added to the Methods section (L85-88).

3. We note that Figure in your submission contain map and images which may be copyrighted. All PLOS content is published under the Creative Commons Attribution License (CC BY 4.0), which means that the manuscript, images, and Supporting Information files will be freely available online, and any third party is permitted to access, download, copy, distribute, and use these materials in any way, even commercially, with proper attribution. For these reasons, we cannot publish previously copyrighted maps or satellite images created using proprietary data, such as Google software (Google Maps, Street View, and Earth). For more information, see our copyright guidelines: <http://journals.plos.org/plosone/s/licenses-and-copyright>

We require you to either (1) present written permission from the copyright holder to publish these figures specifically under the CC BY 4.0 license, or (2) remove the figures from your submission.

Figure 1B is an original map generated in R using the ozmap package and publicly available Australian Bureau of Statistics data. No Google Maps, Google Earth, satellite imagery, or other proprietary mapping imagery was used. We have updated the figure caption (L65–66) to clarify the source data and method used to construct the map.

4. If the reviewer comments include a recommendation to cite specific previously published works, please review and evaluate these publications to determine whether they are relevant and should be cited. There is no requirement to cite these works unless the editor has indicated otherwise.

Not applicable.
