# Supplementary material for: The antibacterial and antifungal activity of honey and propolis from the Australian stingless bee Tetragonula carbonaria
Source: PLoS One. 2026 Oct 26;21(10):e0361610. doi: 10.1371/journal.pone.0361610 (PMC13704853; doi:10.1371/journal.pone.0361610)
Supplement: Peer review history

# Peer review: Decision Letter 0

**DOI:** 10.1371/journal.pone.0361610.r001

**Authors:** Naji Arafat Mahat

30 Jul 2026

Dear Dr. Fernandes,

Thank you for submitting your manuscript to PLOS ONE. After careful consideration, we feel that it has merit but does not fully meet PLOS ONE’s publication criteria as it currently stands. Therefore, we invite you to submit a revised version of the manuscript that addresses the points raised during the review process.

There is a major concern: no chemical characterization of the studied bee products has been performed; only H₂O₂ levels of honey were determined.

Please submit your revised manuscript by Sep 28 2026 11:59PM. If you will need more time than this to complete your revisions, please reply to this message or contact the journal office at plosone@plos.org. When you're ready to submit your revision, log on to https://www.editorialmanager.com/pone/ and select the 'Submissions Needing Revision' folder to locate your manuscript file.

- A letter that responds to each point raised by the academic editor and reviewer(s). You should upload this letter as a separate file labeled 'Response to Reviewers'.
- A marked-up copy of your manuscript that highlights changes made to the original version. You should upload this as a separate file labeled 'Revised Manuscript with Track Changes'.
- An unmarked version of your revised paper without tracked changes. You should upload this as a separate file labeled 'Manuscript'.

If you would like to make changes to your financial disclosure, please include your updated statement in your cover letter. Guidelines for resubmitting your figure files are available below the reviewer comments at the end of this letter.

If applicable, we recommend that you deposit your laboratory protocols in protocols.io to enhance the reproducibility of your results. Protocols.io assigns your protocol its own identifier (DOI) so that it can be cited independently in the future. For instructions see: https://journals.plos.org/plosone/s/submission-guidelines#loc-laboratory-protocols. Additionally, PLOS ONE offers an option for publishing peer-reviewed Lab Protocol articles, which describe protocols hosted on protocols.io. Read more information on sharing protocols at https://plos.org/protocols?utm_medium=editorial-email&utm_source=authorletters&utm_campaign=protocols.

As the corresponding author, your ORCID iD is verified in the submission system and will appear in the published article. PLOS supports the use of ORCID, and we encourage all coauthors to register for an ORCID iD and use it as well. Please encourage your coauthors to verify their ORCID iD within the submission system before final acceptance, as unverified ORCID iDs will not appear in the published article. *Only*  the individual author can complete the verification step; PLOS staff *cannot*  verify ORCID iDs on behalf of authors.

We look forward to receiving your revised manuscript.

Kind regards,

Naji Arafat Mahat, PhD

Academic Editor

PLOS One

Journal Requirements:

When submitting your revision, we need you to address these additional requirements.

1. Please ensure that your manuscript meets PLOS ONE's style requirements, including those for file naming. The PLOS ONE style templates can be found at

https://journals.plos.org/plosone/s/file?id=wjVg/PLOSOne_formatting_sample_main_body.pdf and

https://journals.plos.org/plosone/s/file?id=ba62/PLOSOne_formatting_sample_title_authors_affiliations.pdf

2. In your Methods section, please provide additional information regarding the permits you obtained for the work. Please ensure you have included the full name of the authority that approved the field site access and, if no permits were required, a brief statement explaining why

3. We note that Figure in your submission contain map and images which may be copyrighted. All PLOS content is published under the Creative Commons Attribution License (CC BY 4.0), which means that the manuscript, images, and Supporting Information files will be freely available online, and any third party is permitted to access, download, copy, distribute, and use these materials in any way, even commercially, with proper attribution. For these reasons, we cannot publish previously copyrighted maps or satellite images created using proprietary data, such as Google software (Google Maps, Street View, and Earth). For more information, see our copyright guidelines: http://journals.plos.org/plosone/s/licenses-and-copyright.

We require you to either (1) present written permission from the copyright holder to publish these figures specifically under the CC BY 4.0 license, or (2) remove the figures from your submission:

1. You may seek permission from the original copyright holder of Figure 1 to publish the content specifically under the CC BY 4.0 license.

We recommend that you contact the original copyright holder with the Content Permission Form (http://journals.plos.org/plosone/s/file?id=7c09/content-permission-form.pdf) and the following text:

“I request permission for the open-access journal PLOS ONE to publish XXX under the Creative Commons Attribution License (CCAL) CC BY 4.0 (http://creativecommons.org/licenses/by/4.0/). Please be aware that this license allows unrestricted use and distribution, even commercially, by third parties. Please reply and provide explicit written permission to publish XXX under a CC BY license and complete the attached form.”

Please upload the completed Content Permission Form or other proof of granted permissions as an ""Other"" file with your submission.

In the figure caption of the copyrighted figure, please include the following text: “Reprinted from [ref] under a CC BY license, with permission from [name of publisher], original copyright [original copyright year].”

2. If you are unable to obtain permission from the original copyright holder to publish these figures under the CC BY 4.0 license or if the copyright holder’s requirements are incompatible with the CC BY 4.0 license, please either i) remove the figure or ii) supply a replacement figure that complies with the CC BY 4.0 license. Please check copyright information on all replacement figures and update the figure caption with source information. If applicable, please specify in the figure caption text when a figure is similar but not identical to the original image and is therefore for illustrative purposes only.

The following resources for replacing copyrighted map figures may be helpful:

USGS National Map Viewer (public domain): http://viewer.nationalmap.gov/viewer/

The Gateway to Astronaut Photography of Earth (public domain): http://eol.jsc.nasa.gov/sseop/clickmap/

Maps at the CIA (public domain): https://www.cia.gov/library/publications/the-world-factbook/index.html and https://www.cia.gov/library/publications/cia-maps-publications/index.html

NASA Earth Observatory (public domain): http://earthobservatory.nasa.gov/

Landsat: http://landsat.visibleearth.nasa.gov/

USGS EROS (Earth Resources Observatory and Science (EROS) Center) (public domain): http://eros.usgs.gov/#

Natural Earth (public domain): http://www.naturalearthdata.com/

4. If the reviewer comments include a recommendation to cite specific previously published works, please review and evaluate these publications to determine whether they are relevant and should be cited. There is no requirement to cite these works unless the editor has indicated otherwise.

[Note: HTML markup is below. Please do not edit.]

Reviewers' comments:

Reviewer's Responses to Questions

**Comments to the Author**

1. Is the manuscript technically sound, and do the data support the conclusions?

Reviewer #1: Yes

Reviewer #2: Yes

**********

2. Has the statistical analysis been performed appropriately and rigorously?

Reviewer #1: Yes

Reviewer #2: Yes

**********

3. Have the authors made all data underlying the findings in their manuscript fully available?

The PLOS Data policy

Reviewer #1: Yes

Reviewer #2: Yes

**********

4. Is the manuscript presented in an intelligible fashion and written in standard English?

Reviewer #1: Yes

Reviewer #2: Yes

**********

Reviewer #1: The present manuscript is dedicated to the antibacterial and antifungal potential of honey and propolis from the Australian stingless bee Tetragonula carbonaria. The results are interesting and important. However, there is a major concern: no chemical characterization of the studied bee products has been performed, only H2O2 levels of honey were determined. The Authors should add chemical profiling of the studied products, to make their interesting results much more valuable and scientifically sound.

Reviewer #2: Dear authors,

Please address the following concerns,

1. L93: “Ethanolic extracts were prepared from eight propolis samples from NSW”. Was this referring to intact honey pots sample or filtered honey samples?

2. How many replicated were prepared for H2O2 concentration test?

3. L151: H2O2 ; state in full name for first introduction of the acronym.

4. L159: symbols missing

5. What is the SD for results displayed in Table 2?

6. Figure 1 (B): It is advisable to change into a proper map.

7. Look into typos and grammatical errors meticulously.

**********

what does this mean?). If published, this will include your full peer review and any attached files.

If you choose “no”, your identity will remain anonymous but your review may still be made public.

**Do you want your identity to be public for this peer review?**  For information about this choice, including consent withdrawal, please see our Privacy Policy

Reviewer #1: No

Reviewer #2: No

**********

[NOTE: If reviewer comments were submitted as an attachment file, they will be attached to this email and accessible via the submission site. Please log into your account, locate the manuscript record, and check for the action link "View Attachments". If this link does not appear, there are no attachment files.]

To ensure your figures meet our technical requirements, please review our figure guidelines: https://journals.plos.org/plosone/s/figures

You may also use PLOS’s free figure tool, NAAS, to help you prepare publication quality figures: https://journals.plos.org/plosone/s/figures#loc-tools-for-figure-preparation.

NAAS will assess whether your figures meet our technical requirements by comparing each figure against our figure specifications.

# Author comment: Author response to Decision Letter 1

**DOI:** 10.1371/journal.pone.0361610.r002

19 Sep 2026

We thank the reviewers for their time and constructive feedback which we have used to improve our manuscript. We address each comment below.

Reviewer #1:

The present manuscript is dedicated to the antibacterial and antifungal potential of honey and propolis from the Australian stingless bee Tetragonula carbonaria. The results are interesting and important. However, there is a major concern: no chemical characterization of the studied bee products has been performed, only H2O2 levels of honey were determined. The Authors should add chemical profiling of the studied products, to make their interesting results much more valuable and scientifically sound.

We thank the reviewer for raising this important point, and we agree that chemical characterisation adds valuable context to the interpretation of our antimicrobial findings. We would like to clarify that the present manuscript builds directly on our previous work (https://doi.org/10.1128/aem.02523-24), in which we conducted detailed physicochemical characterisation of honey from T. carbonaria and two other Australian stingless bee species. As this characterisation has already been published by our group and is referenced in the manuscript, we did not consider it necessary to repeat it here (mentioned L70-71).

For propolis, in response to the reviewer's comment we have added chemical profiling by measuring total phenolic content (Folin-Ciocalteu assay) and antioxidant reducing capacity (FRAP assay) and tested these against the observed antimicrobial activity. These additions are reflected in the Abstract (L20-21), Methods (L165-174, L186-190), Results (L242-254) and Discussion (L387-392). We have additionally added a new figure (Figure 3) to present the phenolic content and antioxidant capacity data.

Reviewer #2:

Dear authors, please address the following concerns:

1. L93: “Ethanolic extracts were prepared from eight propolis samples from NSW”. Was this referring to intact honey pots sample or filtered honey samples?

Thank you for flagging this ambiguity. The propolis used for ethanolic extraction was separated from intact honey pot samples. We have updated the Methods to state this explicitly (L98-99).

2. How many replicated were prepared for H2O2 concentration test?

A single measurement was performed for the H2O2 test strip measurements for each honey sample. We have updated the Methods to clarify this (L163-164).

3. L151: H2O2 ; state in full name for first introduction of the acronym.

We have corrected this and now spell out “hydrogen peroxide (H2O2)” at first mention (L152).

4. L159: symbols missing

We appreciate the reviewer flagging this, but we are unable to identify which specific symbols were missing at this line. We have carefully re-checked the manuscript for formatting and symbol errors in general.

5. What is the SD for results displayed in Table 2?

For honey samples with a discrete (i.e. measurable) MIC value in Table 2, the standard error of the mean is shown in Figure 2. Unfortunately, we are not able to calculate a standard devision for samples where the MIC exceeded the highest tested concentration (>25 % w/v), as no numerical endpoint was reached.

6. Figure 1 (B): It is advisable to change into a proper map.

Figure 1B is a geographically accurate map, generated in R using the ozmap package and Australian Bureau of Statistics data. We have updated the figure caption (L65-66) to make the source and method of the map’s construction clearer.

7. Look into typos and grammatical errors meticulously.

We thank the reviewer for this note and have carefully re-proofread the entire manuscript, correcting small typos and grammatical errors.

Journal Requirements:

1. Please ensure that your manuscript meets PLOS ONE's style requirements, including those for file naming.

The manuscript conforms to the PLOS ONE formatting and style requirements.

2. In your Methods section, please provide additional information regarding the permits you obtained for the work. Please ensure you have included the full name of the authority that approved the field site access and, if no permits were required, a brief statement explaining why.

Additional information has been added to the Methods section (L85-88).

3. We note that Figure in your submission contain map and images which may be copyrighted. All PLOS content is published under the Creative Commons Attribution License (CC BY 4.0), which means that the manuscript, images, and Supporting Information files will be freely available online, and any third party is permitted to access, download, copy, distribute, and use these materials in any way, even commercially, with proper attribution. For these reasons, we cannot publish previously copyrighted maps or satellite images created using proprietary data, such as Google software (Google Maps, Street View, and Earth). For more information, see our copyright guidelines: http://journals.plos.org/plosone/s/licenses-and-copyright

We require you to either (1) present written permission from the copyright holder to publish these figures specifically under the CC BY 4.0 license, or (2) remove the figures from your submission.

Figure 1B is an original map generated in R using the ozmap package and publicly available Australian Bureau of Statistics data. No Google Maps, Google Earth, satellite imagery, or other proprietary mapping imagery was used. We have updated the figure caption (L65–66) to clarify the source data and method used to construct the map.

4. If the reviewer comments include a recommendation to cite specific previously published works, please review and evaluate these publications to determine whether they are relevant and should be cited. There is no requirement to cite these works unless the editor has indicated otherwise.

Not applicable.

Attachment Submitted filename: Response to Reviewers.docx

# Peer review: Decision Letter 1

**DOI:** 10.1371/journal.pone.0361610.r003

**Authors:** Naji Arafat Mahat

13 Oct 2026

The Antibacterial and Antifungal Activity of Honey and Propolis from the Australian Stingless Bee Tetragonula carbonaria

PONE-D-26-31337R1

Dear Dr. Fernandes,

We’re pleased to inform you that your manuscript has been judged scientifically suitable for publication and will be formally accepted for publication once it meets all outstanding technical requirements.

Within one week, you’ll receive an e-mail detailing the required amendments. When these have been addressed, you’ll receive a formal acceptance letter and your manuscript will be scheduled for publication.

An invoice will be generated when your article is formally accepted. Please note, if your institution has a publishing partnership with PLOS and your article meets the relevant criteria, all or part of your publication costs will be covered. Please make sure your user information is up-to-date by logging into Editorial Manager at Editorial Manager® and clicking the ‘Update My Information' link at the top of the page. For questions related to billing, please contact billing support.

If your institution or institutions have a press office, please notify them about your upcoming paper to help maximize its impact. If they’ll be preparing press materials, please inform our press team as soon as possible -- no later than 48 hours after receiving the formal acceptance. Your manuscript will remain under strict press embargo until 2 pm Eastern Time on the date of publication. For more information, please contact onepress@plos.org.

Kind regards,

Naji Arafat Mahat, PhD

Academic Editor

PLOS One

Additional Editor Comments (optional):

Reviewers' comments:

Reviewer's Responses to Questions

**Comments to the Author**

Reviewer #1: (No Response)

Reviewer #2: All comments have been addressed

**********

2. Is the manuscript technically sound, and do the data support the conclusions?

Reviewer #1: Yes

Reviewer #2: Yes

**********

3. Has the statistical analysis been performed appropriately and rigorously?

Reviewer #1: Yes

Reviewer #2: Yes

**********

4. Have the authors made all data underlying the findings in their manuscript fully available?

The PLOS Data policy

Reviewer #1: Yes

Reviewer #2: Yes

**********

5. Is the manuscript presented in an intelligible fashion and written in standard English?

Reviewer #1: Yes

Reviewer #2: (No Response)

**********

Reviewer #1: The Authors have answered satisfactorily.

Reviewer #2: Dear authors,

1.L.172: Symbol missing.

2.Suggested to include Figure 4 and Figure 5 in text.

Authors had addressed all my previous comments.

**********

what does this mean?). If published, this will include your full peer review and any attached files.

If you choose “no”, your identity will remain anonymous but your review may still be made public.

**Do you want your identity to be public for this peer review?**  For information about this choice, including consent withdrawal, please see our Privacy Policy

Reviewer #1: No

Reviewer #2: No

**********

# Editor report: Acceptance letter

**DOI:** 10.1371/journal.pone.0361610.r004

**Authors:** Naji Arafat Mahat

PONE-D-26-31337R1

PLOS One

Dear Dr. Fernandes,

I'm pleased to inform you that your manuscript has been deemed suitable for publication in PLOS One. Congratulations! Your manuscript is now being handed over to our production team.

At this stage, our production department will prepare your paper for publication. This includes ensuring the following:

* All references, tables, and figures are properly cited

* All relevant supporting information is included in the manuscript submission,

* There are no issues that prevent the paper from being properly typeset

You will receive further instructions from the production team, including instructions on how to review your proof when it is ready. Please keep in mind that we are working through a large volume of accepted articles, so please give us a few days to review your paper and let you know the next and final steps.

Lastly, if your institution or institutions have a press office, please let them know about your upcoming paper now to help maximize its impact. If they'll be preparing press materials, please inform our press team within the next 48 hours. Your manuscript will remain under strict press embargo until 2 pm Eastern Time on the date of publication. For more information, please contact onepress@plos.org.

You will receive an invoice from PLOS for your publication fee after your manuscript has reached the completed accept phase. If you receive an email requesting payment before acceptance or for any other service, this may be a phishing scheme. Learn how to identify phishing emails and protect your accounts at https://explore.plos.org/phishing.

If we can help with anything else, please email us at customercare@plos.org.

Thank you for submitting your work to PLOS One and supporting open access.

Kind regards,

PLOS One Editorial Office Staff

on behalf of

Dr. Naji Arafat Mahat

Academic Editor

PLOS One
